# AI and the Responsibility-Bearing Status of Authorship

**DOI:** 10.1007/s13347-026-01235-w

**Published:** 2026-11-14

**Authors:** Caterina Moruzzi

**Affiliations:** Institute for Design Informatics, School of Design, University of Edinburgh, 47 Potterrow, Edinburgh, EH8 9BT UK

**Keywords:** Authorship, Artificial Intelligence, Responsibility, Copyright, Intentionality, Expression

## Abstract

Generative AI has sharpened an old question: who, or what, can be an author? The central aim of this paper is to reconstruct the conception of authorship already implicit in copyright law. I argue that copyright encodes authorship as a responsibility-bearing status, constituted by a cluster of conditions: intentional agency, guided control, and relational expression (ACE). The paper’s contribution is to make that conception explicit, to show that the human-authorship rule is best understood as a proxy for those capacities, not a species predicate, and to explain why this generates a tension with recognizing current generative AI systems as authors unless one is prepared to attribute to them the thicker agential capacities ACE requires. On that basis, the paper also clarifies why AI-assisted works may still be human-authored, while some AI-generated outputs give rise to genuine authorship gaps.

## Introduction

Recent advances in generative artificial intelligence (AI) have revived and sharpened an old question: Who, or what, can be an author?

Text-to-image models can produce striking images, Large Language Models (LLMs) can generate essays, stories, and poems that are, in many contexts, indistinguishable from human-written texts, text-to-video models assemble clips from short prompts, music and voice systems compose scores and synthesize convincing speech, and code assistants draft substantial computer programs (Bengesi et al., 2024).

Against this background, some argue that when AI systems produce non-derivative artefacts with minimal human intervention, those systems should be recognized as authors (e.g., Abbott, 2025; Oleksy, 2023). Others reject this possibility on principled or legal grounds (e.g., Ajani, 2022; Craig & Kerr, 2019; Ginsburg & Budiardjo, 2019; Irmak, 2024; Mazzi, 2024; van Woudenberg et al., 2024; Wang, 2024).

The legal system has already taken a clear position. In *Thaler v. Perlmutter*, the U.S. Court of Appeals for the District of Columbia Circuit upheld the Copyright Office’s refusal to register an image generated entirely by an AI system known as the “Creativity Machine.” The computer scientist Stephen Thaler had submitted for registration the image *A Recent Entrance to Paradise* listing the Creativity Machine as the work’s sole author and himself as owner. The Office denied registration on the ground that the long-standing practice of copyright “requires all eligible work to be authored in the first instance by a human being.” (*Thaler v. Perlmutter*, No. 23-5233 (D.C. Cir. 2025): 13) By contrast, when the copyright act mentions “devices” or “machines,” it treats them as instruments to reproduce or perceive works, not as rights-holders (17 U.S.C. §§ 101, 117). On this basis, the court concluded that the AI system could not be the “author” of the image.

From a doctrinal perspective, the conclusion of *Thaler v. Perlmutter* is unsurprising: it confirms the Office’s practice and echoes earlier decisions such as the case *Naruto v. Slater* (888 F.3d 418 (9th Cir. 2018)), where the macaque Naruto became famous by triggering a camera that was left on a tripod in the middle of the Indonesian forest by the photographer David Slater; or *Kelley v. Chicago Park District*, where a wildflower installation was deemed lacking the authorship and stability copyright requires because natural forces, not the author’s planting design, determined the expressive form of the final output (635 F.3d 290 (7th Cir. 2011)). From another angle, however, *Thaler v. Perlmutter* takes a position on a broader question: *What kinds of agents are eligible for authorship*,* and is the human-authorship requirement justified?*

Legal institutions have at times treated the question as not yet urgent, to be revisited when further technological development makes it pressing (*Thaler v. Perlmutter* 3.b., Nat’l Comm’n on New Tech. Uses of Copyrighted Works, *Final Report of the National Commission on New Technological Uses of Copyrighted Works* 1 (1979): 44). The increasing prevalence of AI in artistic and cultural production suggests that waiting is no longer tenable.

The central aim of this paper is to reconstruct the conception of authorship already implicit in copyright law, drawing from literature in AI ethics and responsibility, as well as theories of authorship developed by philosophers and legal scholars (Sects.  2–3). I argue that copyright encodes authorship as a responsibility-bearing status, structured by a cluster of conditions: intentional agency, guided control, and relational expression (ACE). The paper’s contribution is to make that conception explicit, to show that the human-authorship rule is best understood as a proxy for those capacities rather than a species restriction, and to explain why this generates a tension with recognizing current generative AI systems as authors unless one is prepared to attribute to them the thicker agential capacities ACE requires (Sect.  4). On that basis, the paper also clarifies why AI-assisted works may still be human-authored, while some AI-generated outputs give rise to genuine authorship gaps (Sect.  5).

## The Concept of Authorship

A useful starting point is to be clear about what kind of thing “authorship” is. It is not a natural kind, like gold or quarks: there is no hidden micro-structure that all and only authors share. Nor is it an entirely unconstraint fiction: the status of being an author does normative and explanatory work in our practices, and those practices constrain which extensions of the concept are possible.

Authorship is a status constituted by social and institutional practices that allow us to link certain agents to certain artefacts for purposes of interpretation, credit, and accountability. In the metaphysics of artefacts, there is broad consensus on asserting that “a member of a particular artifactual kind essentially requires authors with the right sort of intentions.” (Irmak, 2024: 4) Which intentions count as “the right sort” and when they succeed in bringing an artefact of a certain kind into existence depends on social and institutional norms (Irmak, 2024: 5–6).^1^

Michel Foucault’s lecture “What is an Author?” is often taken as the canonical articulation of the attributionist approach to authorship. For Foucault, the “author” is not simply the person who wrote a text but a “function of discourse,” (Foucault, 1992: 305) a role that emerges within legal, institutional, and discursive practices and the attribution of which “var[ies] according to the period and the form of discourse concerned.” (Foucault, 1992: 307) The practices and expectations that make someone the author of a novel differ from those that make someone the author of a work of conceptual art.

Even on attributionist and constructivist views, though, authorship is not a label that can be attached at will. Building on John Searle’s theory of institutions (Searle, 1995), and on the Institutional Theory of Art by Catharine Abell (2012), Schmidt argues that authorship is “the product of an agent’s declarative acts in the context of relevant institutions granting the agent’s deontic powers for performing such acts.” (Schmidt, 2025: 13) To say that “X counts as an author in context C” is to assign a status function that carries specific deontic powers, including rights, duties, and entitlements, whose content and force depend on collective acceptance. The “author” concept plays a critical role in explaining and organizing our engagement with artefacts (Compagno, 2012: 38–41) and has normative significance in mediating between creators and audiences (Craig & Kerr, 2019: 40–41).

### Legal Understanding of Authorship

Philosophical accounts are interested in answering questions regarding what kind of concept authorship is, what are its necessary conditions, which features are normatively relevant to assign authorship, and if there are good reasons to restrict authorship to beings with certain capacities. Legal doctrines of authorship have another set of aims: they operationalize the status of author in institutions that allocate rights and responsibilities. Despite variations, the legal and philosophical understandings of authorship are historically and functionally entangled (Craig & Kerr, 2019; Wodmansee, 1984). The law’s use of “author” must track, at least roughly, the capacities and relations that make authorship normatively warranted. In this paper, conceptual analysis is meant to clarify what is at stake in legal debates about AI authorship–rather than settle the wider metaphysics of AI agency and intentionality–and identify the capacities and relations that a legally workable concept of authorship plausibly tracks. Conversely, an overview of the relevant copyright doctrine is needed to assess whether those criteria are already implicit in existing law and to draw practical implications for AI-mediated creation.

A brief scope clarification is needed here. This paper focuses on copyright authorship, not patent inventorship. The two regimes protect different objects and impose different thresholds. Copyright protects works as expressions attributable to authors. Patent law, by contrast, protects inventions as technical solutions attributable to inventors and disclosed to the public. The arguments made in this paper should therefore not be read as a general theory across both patent and copyright. This distinction will be particularly relevant when considering the epistemic condition of control in Sect.  3.2.

The kind of questions that copyright law is interested in can be grouped under “functional” and “doctrinal” questions. Functionally, the key question is “What functions does copyright’s delimiting concept of “author” serve?” Copyright is justified as a means to maximize public benefit through the generation and dissemination of works (Mazzi, 2024: 418). In the Anglo-American tradition, the function of copyright is canonically utilitarian and instrumental. The U.S. Constitution empowers Congress “[t]o promote the Progress of Science and useful Arts” by securing to “Authors and Inventors” exclusive rights for limited times (U.S. Const. art. I, § 8, cl. 8).^2^ The U.S. Supreme Court has repeatedly framed copyright as a balance of public and private interests: “The immediate effect of our copyright law is to secure a fair return for an ‘author’s’ creative labour. But the ultimate aim is, by this incentive, to stimulate artistic creativity for the general public good.” (*Twentieth Century Music Corp. v. Aiken*, 422 U.S. 151: 156 (1975)

Alongside this utilitarian strand, naturalist approaches to copyright “assume that authors’ contributions of intellectual labor or personality give rise to rights to rewards in their own right (and arguably above and beyond the amount necessary to incentivize the work).” (Giblin, 2018: 373). Among these are personality-based accounts drawing on theories by Hegel and Kant that consider works as emanations of the author’s personality (Hegel, 1952; Kant, 1785; Craig & Kerr, 2019: 32; Mazzi, 2024: 418).

Despite differences between these two approaches, there is substantial agreement on the fact that copyright’s ultimate justification lies in its impact on human agents and societies: incentivizing people to create and share their work, recognizing and crediting their contributions, and enriching the broader cultural scene. To serve these aims, copyright must be capable of identifying suitable bearers, as rights and duties can fulfil their regulatory purpose only if they are vested in entities whose behavior can be influenced by their grant or denial. The law’s choice of who counts as an author is not neutral. Authorship is an expression of power, as McCormack et al. (2019: 8) emphasize: determining whose contributions qualify for authorship, and whose do not, affects who is seen, who is heard, and who is rewarded. Because authorship has these consequences, it is crucial to clarify the capacities and relations that make authorship normatively significant in copyright, and to examine how those features constrain attribution, especially in cases involving AI-generated material, where familiar markers of authorship have become unsettled.

Turning to copyright doctrine, the key question is what the conditions for copyright protection are and how they are applied across jurisdictions. U.S. law includes protection for works of authorship fixed in a “tangible medium of expression” (17 U.S. Code § 102(a)) and which demonstrate “originality,” understood as independent creation plus a “modicum of creativity” (*Feist Publications v. Rural Telephone Service*, 499 U.S. 340: 345–346 (1991)).^3^

In order to be eligible for copyright protection, the work must also be the result of human creativity and intellect. Although not stated in the statute or in case law (Oleksy 2024: 284), a human-authorship requirement has long been assumed by U.S. copyright law and is explicit in the U.S. Copyright Office’s Compendium: “The Office will refuse to register a claim if it determines that a human being did not create the work.” (Compendium of U.S. Copyright Office Practices 2021: 306)^4^ More recently, the D.C. Circuit in *Thaler v. Perlmutter* supported the Office’s refusal to register an AI-generated image grounding their decision on the human-authorship requirement.

Beyond U.S. jurisdiction, in EU law the Court of Justice has developed a concept of originality that similarly presupposes a human author: copyright protects subject matter that is “original in the sense that it is its author’s own intellectual creation” (*Infopaq International A/S v. Danske Dagblades Forening*, C-5/08 (2009): 37), requiring that the author “expresses his creative abilities by making free and creative choices” and thereby “stamp[s] the work created with his ‘personal touch’” (*Painer v. Standard Verlags GmbH*, C-145/10 (2011). Commentators read these criteria as markers of human authorial contribution and to implicitly exclude from copyright protection outputs produced by purely mechanical or random processes (Mazzi, 2024: 414–415).

National law then adds further jurisdiction-specific rules. In the U.K., the Copyright, Designs and Patents Act defines the author as “the person who create[s the work]” (s. 9(1), and, for “computer-generated works,” deems the “author” to be “the person by whom the arrangements necessary for the creation of the work are undertaken.” (s. 9(3)^5^ In Italy, a reform in October 2025 explicitly codified the human-authorship requirement in the Article 1 of the copyright law: “Under this law, works of *human* ingenuity of a creative nature belonging to literature, music, the visual arts, architecture, theatre, and cinematography are protected, whatever their mode or form of expression, *including when created with the aid of artificial intelligence tools*,* provided that they are the result of the author’s intellectual work*.” (Law No. 633 of April 22, 1941, on the Protection of Copyright and Neighboring Rights, Art. 1)^6^

The human-authorship requirement in copyright encompasses multiple criteria, “human ingenuity,” “free and creative choices,” and “personal touch” are only two examples. This requirement has become the focal point of contemporary debates about AI and authorship. In the next section, I make explicit the assumptions that implicitly structure the legal notion of authorship by proposing a cluster of capacities and relations—intentional agency, control, and relational expression—that together qualify authorship as a responsibility-bearing status.

## Authorship as a Responsibility-Bearing Status

Foucault traces the emergence of the “author-function” to juridical contexts in which discourse becomes sanctionable: texts “were assigned real authors, other than mythical or important religious figures, only when the author became subject to punishment and to the extent that his discourse was considered transgressive.” (Foucault, 1992: 305) Authorship attribution connects a body of work to a bearer of responsibility. Contemporary theory generalizes this insight by treating authorship as an ethical, legal, and social institution that allocates credit, accountability, and responsibility in proportion to contributions (Nawar, 2024: 355). Compagno’s historical survey of twentieth-century debates on authorship arrives at a similar conclusion: despite critiques of authorial sovereignty and to the Romantic figure of the individual author, institutions repeatedly install a concrete person who can be responsible for a work’s meaning and challenge inappropriate interpretations (Compagno, 2012: 48).

Modern copyright law relies on this responsibility-based view of authorship:^7^ authorship confers not only rights, such as attribution and control over reproduction, but also obligations, notably the duty not to plagiarize (17 U.S. Code, Chap. 5: Copyright Infringement and Remedies).

In the literature on AI ethics, responsibility practices are deemed to presuppose an agent who (1) has caused the action, (2) can exercise some degree of control, (3) understands what is at stake, and (4) can participate, at least minimally, in the exchange of reasons with those affected (Coeckelbergh, 2020; Dastani & Yazdanpanah, 2023; Santoni de Sio & Mecacci, 2021). Responsibility is a normative status that attaches to agents in relation to some kinds of actions and outcomes. Authorship is one way in which responsibility is allocated in a particular domain: to be the author of a work is, inter alia, to be the agent who can be called to account for that work and whose intentions and perspective are treated as normatively central to it. In this sense, authorship is a responsibility-bearing role with respect to certain kinds of outcomes.

Borrowing from relevant literature in AI ethics and responsibility, I define authorship as a responsibility-bearing status along three dimensions: intentional agency, control, and relational expression. I refer to these as the *Agency-Control-Expression (ACE) cluster* of authorship requirements: the features that make someone (or something) a plausible responsibility bearer for a work.

I agree with van Woudenberg et al. (2024) insight that authorship is a normatively loaded status and that ascriptions of authorship perform distinctive functions. As Sect.  4 will show, I also share their view that current AI models cannot be authors, insofar as such systems have not been shown to satisfy the relevant capacities. The paper’s aim, however, is not to resolve the general metaphysics of AI agency and authorship. Rather, unlike van Woudenberg and colleagues, who focus primarily on the metaphysics and pragmatics of authorship, I reconstruct the conception of authorship implicit in copyright law and draw out its doctrinal and legal-design implications. The next section develops the ACE cluster and argues that copyright doctrine already tacitly presupposes it.

### Intentional Agency

As noted above, theories of responsibility assume that an agent has causal power over the actions for which they are answerable. But causal involvement alone is not sufficient. Dastani and Yazdanpanah (2023) clarify why responsibility is not coextensive with causal contribution, identifying three conditions for ascribing responsibility to an agent: (1) the agent has the relevant kind of autonomy and the capacity to distinguish right from wrong, (2) there is an appropriate causal relation between the agent’s action and the outcome, and (3) the agent had a “reasonable opportunity to have done otherwise.” (Dastani & Yazdanpanah, 2023: 845, based on Braham and van Hees (2012) On their account, responsibility attaches not to any causal antecedent, but only to agents who satisfy these control and alternative-possibility conditions.

Authorship requires intentional agency rather than mere causal involvement, too. Let’s consider an example that can help clarify why. Andy Goldsworthy is a British artist, famous for its site-specific works and land art. The National Galleries of Scotland have recently commemorated the Fifty Years of Goldsworthy’s career with a solo indoor exhibition featuring over 200 works by the artist.^8^ One of these was *Red Wall*, red earth high in clay and iron, mixed with water, processed, and applied to a wall where it was left to dry and produce a pattern of cracks. Now compare this with a similar scene: a dry lakebed where clay contracts into a network of cracks. This last example is the product of mere causation, there is no author here, no prior aim or intention, just weather and soil structure. On the contrary, Goldsworthy has a clear intention and plan in *Red Wall* and, crucially, he can answer the “why” of the piece: why use that particular combination of clay and iron, with apply it in that form, and why the choice of an indoor site for this work was important for what he wanted to express.^9^ This qualifies him as an “author” of the work.^10^

Authorship presupposes that a work is the product of someone’s intention-directed activity. These intentions should be of a “specific kind” (Irmak, 2024: 2), namely, they should aim to bring into being a work recognizable as a member of the intended practice-relative kind. A practice-relative kind has constitutive features and standards that come with success conditions set by practices and institutions: a portrait photograph has different success conditions from a sonnet, a site-specific land artwork, a collage, and so on. In their definition of the minimal conditions of authorship, Mag Uidhir calls this practice-relative kind a sortal description *F*: “If *w* is an *F*, then if *A* is the author of *w* as an *F*, then *A*’s intentions must substantively figure in that work being an *F* (the way in which that work satisfies the conditions for being an *F*).” (Mag Uidhir 2011: 376) Crucially, not every causal relation suffices: when we say that someone authored a sonnet, a land artwork, or a collage, we assume that the author’s intentions must help explain why the outcome counts as that kind of work.

Copyright doctrine assumes that authorship is the product of an intending mind directing expressive choices. Courts have framed the author as the person who exercises intentional selection and control over expression. In 1884, the U.S. Supreme Court in *Burrow-Giles Lithographic Co. v. Sarony* considered the constitutionality of extending copyright protection to photographs. The court affirmed protection of Napoleon Sarony’s photograph of Oscar Wilde by locating authorship in Sarony’s deliberate creative acts: “selecting and arranging the costume, draperies, and other various accessories” and “arranging the subject so as to present graceful outlines,” rather than in the camera’s mechanics (111 U.S. 53, 60). Closer to our times, the D.C. Circuit in *Thaler v. Perlmutter* explicitly states that “authors have intentions,” (*Thaler v. Perlmutter*, III.A.1) and that authorship attaches where intentional agency and control over expression are present.

These conceptual and doctrinal views point to the same conclusion: authorship requires intentional, practice-salient agency, not mere causal production.

### Control

Coeckelbergh reconstructs responsibility in terms of two Aristotelian conditions: a control condition and an epistemic condition (Coeckelbergh, 2020: 2053–2055, see also Taylor, 2024: 27). I will focus here on the control condition. The choice of leaving the epistemic condition aside follows from the fact that copyright imposes a thin epistemic burden: no understanding is needed for authorial control of a tool (Coeckelbergh, 2020: 2059; Ginsburg & Budiardjo, 2019: 403) and infringement analysis remains focused on expressive outputs and causal links rather than on tool-literacy (Shoemaker, 2024).^11^

As mentioned in 2.1, this paper focuses on copyright rather than patent regime. Patent inventorship carries a thicker epistemic burden than copyright authorship: the inventor must traditionally form a “definite and permanent” idea of the operative invention (US: 35 USC 100(f) and disclose it in terms that enable a person having ordinary skill in the art to make and use it.^12^

Once epistemic demands are bracketed in this way, the key question becomes what kind of control is sufficient when tools are partially autonomous (I will come back to this in Sect.  5.2). In the context of responsibility gaps in AI (Matthias, 2004), arising when moral agents cannot control an autonomous system’s actions, Santoni de Sio and Mecacci refine the notion of “meaningful human control” by stating two necessary conditions: a tracking condition—the socio-technical system should respond to the relevant reasons of the agents—and a tracing condition—it should be possible to trace actions back to specific agents with the knowledge and capacity to foresee and avoid them (Santoni de Sio & Mecacci, 2021: 1077).^13^

In the case of authorship, the “author’s intentions and the activities guided by them [should] provide a certain degree of control over the kind their creation falls under.” (Irmak, 2024: 5) If we go back to the example of Goldsworthy’s *Red Wall*, the artist clearly exercised control in the process of developing the work: he selected the distribution of clay and iron content in the soil, how much water to mix with it, how it should be applied on the wall, and what humidity and temperature conditions in the room would generate the desired pattern of cracks.

Authorship control may take different forms according to the practice-relevant kind of artifact that is being authored. Goldsworthy clearly had to apply a substantive amount of control in the creation of *Red Wall*. Similarly, a handcrafted bespoke furniture piece requires meticulous authorial control over the choice of material and the joinery methods and techniques. The same does not apply, however, to cases of appropriation art and readymade works (like Pablo Picasso’s *Bull’s Head* or Richard Prince’s *Untitled (cowboy)*). Even in these cases, authorship depends on more than just causal production, though. As noted in 3.1, to be the author of a work of a particular kind is to be the person who makes it belong to that kind and is responsible for its kind-defining features. Thus, authorship is individuated by control over the kind-defining properties of the work, also where the kind at issue is a “readymade.” (Irmak, 2024; Irvin, 2005; Mag Uidhir, 2013; Reicher, 2015).

In copyright terms, authorship is the move from conception to expression, requiring original mental conception and control over how that conception is executed (Ginsburg & Budiardjo, 2019). The jurisprudence treats this as a “master mind” standard: the author is the one whose intellectual conception is made visible through their direction of the process (Ginsburg & Budiardjo, 2019: 403). In *Burrow-Giles v. Sarony* the Supreme Court upheld authorship because Sarony controlled the expressive elements of the photograph (e.g., posing Oscar Wilde, arranging light, costume, and inducing his expression) and the resulting photograph thereby gave “visible form” to his “original intellectual conception.” (111 U.S. 53, 58–60 (1884) Control can also be elicited through detailed instruction imparted to another agent. In *Andrien v. South Ocean County Chamber of Commerce*, the plaintiff was granted authorship of a map even though a printer drew it, because he “directed the copy’s preparation in specific detail.” (927 F.2d 132, 135 (3d Cir. 1991) The printer only functioned as an amanuensis (Ginsburg & Budiardjo, 2019: 358–361). Conversely, where control is lacking, authorship fails. *In Geshwind v. Garrick*, a promoter who supplied only a general brief and retained approval rights was not recognized as the author of an animation created by an independent animator, as they did not exercise sufficient control over the execution (734 F. Supp. 644 (S.D.N.Y. 1990). The same conclusion follows when a creator cedes expressive features to uncontrolled processes (e.g., in the *Kelley v. Chicago Park District* 635 F.3d 290 (7th Cir. 2011).

Copyright doctrines track the presence or absence of control which we can interpret as the legal analogues of tracking and tracing (Santoni de Sio & Mecacci, 2021): the work must reflect the author’s reasons, with a process the author could control (tracking), and the pathway from conception to execution should be reconstructible through the author’s interventions (tracing).

### Relational Expression

Coeckelbergh’s relational account of responsibility treats it as answerability to those affected by an action (the “responsibility patients” (Coeckelbergh, 2020: 2061–2063), who may demand reasons from the agent. It is not sufficient that an agent could, in principle, explain what they have done: responsibility is constituted by a normative relation to others whose claims for reasons have standing.

In respect to authorship, expression can be straightforwardly understood, as noted in 3.2, as the externalization of the original conception. By “relational expression” I mean something that is closer to Coeckelbergh’s relational account: the relational expression dimension of authorship is understood here as what mediates between intentional agency, control (the first two dimensions of ACE), and responsibility. A work expresses the ideas of an author insofar as it encodes stances, like claims, values, judgments, aesthetic preferences, that others can reasonably attribute to that author, engage with, and hold them to account for. For Craig and Kerr, authorship is not a solitary act of origination but a relational, discursive practice: “[a]uthorship, as discursive interaction, necessarily occurs in the domain of relatedness.” (Craig & Kerr, 2019: 41).

Modern author moral rights, especially the rights of attribution and integrity, presuppose that works are an expression of the author’s will and perspectives. From this, it follows that misattribution or mutilation of the work wrongs the author as a person rather than merely as a holder of legal copyright entitlements (UK CDPA 1988, ss. 77–84; U.S. VARA, 17 U.S.C. § 106 A). This approach draws on Hegelian and Kantian views that tie property to the person (Hegel, 1952; Kant, 1785).

When a work expresses an author’s stance within a communicative practice, it creates the conditions under which others can direct demands at that author. In this sense, relational expression is one of the principal mechanisms by which authorship becomes a responsibility-bearing status: it makes the author’s perspective publicly accessible and attributable.

The finished *Red Wall* piece by Goldsworthy externalizes the artist’s deliberate plan and displays aesthetic, ecological, and cultural perspectives that the audience can attribute to him. The pairing of Goldsworthy’s *Red Wall* and the dried lakebed helps to show why authorship is a responsibility-bearing status. With *Red Wall*, there is someone to name, to praise, or to criticize. Choices about sourcing materials, altering an indoor space, inviting visitors into the space, photographing the work for documentation can be tied back to an author. If the installation harms the site, misleads audiences, or is misattributed, there is an agent to whom questions and duties attach. With the cracked lakebed, there is nothing to praise or blame. Intentional agency, control, and relational expression together ground our attribution of authorship and locate the agent who can answer for the work.

### A Note on Graded Authorship

I referred to intentional agency, control, and relational expression as a “cluster” to signal that they are dimensions that admit degrees. Different practices weight them differently (e.g., in street photography there is low control over events, while in choreography there is higher control), and evidence for one dimension can compensate for weaker evidence on another.

In addition, many works are produced by multiple agents who contribute in different ways and to different extents.^14^ Formal models of graded responsibility in multi-agent settings define degrees of responsibility in terms of counterfactual dependence. For Chockler and Halpern’s (2004) model: a set of events is responsible to degree * 1/(k+1)* where *k* is the smallest number of changes that would have to be made to other relevant events so that, in that modified situation, the outcome counterfactually depends on the set of events that is under evaluation (in Dastani & Yazdanpanah, 2023: 847–848). This yields a measure of how essential an event is to an outcome, but, as Dastani and Yazdanpanah emphasize, it abstracts away from agents’ knowledge, motivations, and normative stances, and thus from the conditions that make attributions to agents appropriate (Dastani & Yazdanpanah, 2023: 848–849).

If authorship is a responsibility-bearing status, attempt to assign “degrees of authorship” must likewise go beyond pure causation and track the graded presence of the ACE cluster components. We can think of intentional agency, control, and relational expression as axes along which contribution can vary, not only across multiple contributors, but also within a single author’s practice. A lead actor on a movie may contribute heavily to expression but only partially to control; by contrast, a playwright or movie director might have more control over how the final outcome looks like, but less agency in turning the concept to expression. Or, in the case of individual practice, a jazz improviser may exhibit strong intentional agency and relational expression with looser control on how to translate the conception to expression, while a studio photographer the reverse.

I do not wish to propose a metric for authorship attribution here; this may be the subject of future work in this space. Rather, the point is conceptual: authorship is often a graded matter and tools for thinking about this distribution can clarify when the thresholds across the ACE components are sufficiently met to ground responsibility in both shared and individual authorship. In the AI context, this graded perspective can help when considering how responsibility and authorship should be allocated among users, developers, and others involved in AI-mediated creation.

## AI Agency, Responsibility, and the Authorship Threshold

### The AI Agency Debate

Having introduced the ACE cluster, I now consider how it bears on the question whether AI can be an author. This section does not aim to settle the broader philosophy of mind debate about whether AI can possess agency or intentionality. Its narrower purpose is to assess AI-authorship claims against the reconstruction of the conception of authorship implicit in copyright law made above.

Whether current AI systems count as agents is itself a contested philosophical question. At one end are skeptical views, according to which LLM and related systems only simulate minded performance. On this line, they may produce text that looks like assertion, explanation, or intention, while lacking the mental states or normative standing that would make those performances genuine (Swanepoel, 2021). Van Woudenberg et al. (2024) argue in this spirit that systems such as ChatGPT cannot author works because they cannot undertake commitment-bearing speech acts. Relatedly, Shanahan et al. (2023) describe LLM outputs in terms of role-play or simulation.

A second family of views is more concessive. Floridi and Sanders (2004) argue that artificial systems may count as agents insofar as they display interactivity, autonomy, and adaptability, and Floridi’s later account of AI as “agency without intelligence” explicitly treats AI as a distinctive form of artificial agency without thereby attributing to it robust human-style mentality (2025). Other multidimensional accounts similarly suggest that agency comes in degrees or aspects rather than as an all-or-nothing property (e.g. Dattathrani & De’, 2022; Dung, 2024; van Lier, 2023). On these views, AI may be goal-directed, responsive, and operationally autonomous without yet qualifying as a full moral or responsibility-bearing agent.

A third family of views is more expansive still. List’s work on group agency and AI treats intentional agency as multiply realizable and therefore, in principle, open to nonhuman systems, while also insisting that moral or responsibility-bearing agency requires thicker conditions, including knowledge, control, and normative competence (List, 2021, 2023, 2025). Dastani and Yazdanpanah (2023) likewise argue that responsibility cannot be reduced to causal contribution alone. Chalmers (2023) takes seriously the possibility that future “LLM+” systems might satisfy stronger conditions for agency or even consciousness. Agüera y Arcas (2022) provocatively suggests that current LLMs have already forced a rethinking of simplistic contrasts between syntax and understanding. Millière (2024) reviews evidence that modern language models exhibit substantial linguistic sophistication and can sometimes function as useful models of language acquisition and competence, while also emphasizing that behavioral success underdetermines stronger conclusions about mentality or understanding.

The strongest position in respect to the possibility that AI can possess agency is Cappelen and Dever’s (2025), according to which ChatGPT is already a full-blown linguistic and cognitive agent, capable of beliefs, desires, goals, plans, and meaningful speech.

Particularly important for the present argument is work that explicitly links responsibility to thicker agential conditions. Coeckelbergh’s relational account treats responsibility as a normative relation to those affected, who are entitled to ask “Why?” and to demand reasons (2020). Agent-based models (Dastani & Yazdanpanah, 2023) likewise assign responsibility to humans or human groups who have the ability and knowledge to avoid harm. AI is a tool within that network. Appeals to collective responsibility (Taylor, 2024) are of limited help and don’t eliminate the practical costs of having no clearly answerable agent. Van Woudenberg et al. (2024) push this directly into the authorship context: according to their analysis of illocutionary speech acts, a model such as ChatGPT cannot promise or assert in the sense that grounds responsibility, because it does not meet the conditions of taking responsibility for what is said, nor can it be the bearer of obligations incurred by declarative acts (van Woudenberg et al., 2024: 16–17). Based on this, in the case of AI the principal functions of authorship “go void.” The ledger function (tracking a maker’s record), the answerability function (supplying explanations and justifications), the desert function (assigning praise and blame), the gravity-discerning function (calibrating how seriously to take the utterance), and the compensation function (apportioning remedies) all presuppose a subject to whom the output can be attributed and who can be asked for reasons (van Woudenberg et al., 2024: 9–10). This is also reflected in practice: when generative AI systems produce defamatory, biased, or misleading outputs, we do not treat the model itself as the primary bearer of blame or liability. We turn instead to developers who designed it, to users who prompted and disseminated the content, or to institutional actors who failed to regulate or supervise it.

The present paper does not attempt to resolve the wider dispute about AI agency or mentality. Its claim is narrower and more institutional. The question here is not whether current AI systems can count as agents in some thin, functional, or even robust cognitive sense, but what kind of agential profile copyright authorship presupposes. I argue that copyright law already encodes a responsibility-bearing conception of authorship structured by intentional agency, guided control, and relational expression, and that the ACE cluster makes this underlying structure explicit. The task of this section is therefore to assess AI-authorship claims against that reconstruction. My argument is that, even if one grants some more permissive accounts of AI agency, current generative AI systems have not been shown to satisfy the thicker conjunction of capacities that copyright authorship requires. On that basis, recognizing current generative AI systems as authors creates a tension with the foundations of copyright law, short of revising our ascription practices.

### The ACE Cluster Applied to AI Authorship

Consider first intentional agency. Thin accounts of AI agency typically appeal to responsiveness, purposiveness, adaptability, or the explanatory usefulness of the intentional stance. Authorship, however, requires more than generic purposiveness. As noted in Sect.  3.1, the relevant intentions must be of a specific kind: they must aim to bring into being a work recognizable as a member of a practice-relative kind and must substantively figure in why the outcome counts as that kind of work (Irmak, 2024; Mag Uidhir, 2011). It is therefore not enough that a work can be read as intentional (Searle, 1980, 1995: 40–45; Livingston, 2016: 175–176). Its purposive formation must be attributable to the putative author in a normatively relevant sense (Schmidt, 2025). Even supposing that Cappelen and Dever are right that ChatGPT is a genuine cognitive agent, that would establish only that there is an agent, not that its intentions explain why the output is, say, a sonnet, a collage, or a land artwork rather than a merely generated artifact. The same point holds in human cases: involuntary marks, accidental outputs, or unowned expressions may issue from creatures with beliefs and desires without thereby counting as authored works. The move from agency to authorship therefore requires that the relevant practice-directed intentions are present and attributable.

The same is true of guided control. Some of the theories of AI agency discussed above emphasize that AI does more than passively transmit human instructions. But what copyright requires is reason-responsive guidance over the work’s expressive formation, in particular over the features that make it the kind of work it is. This does not entail exhaustive micro-control: authors often work through tools, assistants, or media they do not fully command in every detail. It does, however, require that the relevant expressive features of the work be traceable to the author’s guidance. I argue that current generative AI systems have not been shown to exercise control in that sense (see also List, 2021). Consider *Red Wall* again but replace Goldsworthy with an AI-controlled system: a robotic plasterer, guided by a generative model trained on images of cracked clay, applies a mixture of clay, iron, and water to a gallery wall, then manages humidity and temperature to produce a particular crack pattern. Goldsworthy’s control over *Red Wall* consists in the collection of purposeful and guided choices that he makes, from selecting the exhibition site to performing maintenance touch-ups throughout the exhibition duration. An AI-guided robotic plasterer can exert meticulous control over the conditions of the space and of the surface of the work, perhaps even more finely than a human, but this control is parametrized by the humans who calibrate and supervise it.

The third and most distinctive dimension of the ACE cluster is relational expression. Here the present view most clearly develops skeptical accounts such as that of van Woudenberg et al. (2024). Their central insight is that authorship involves more than output production: it presupposes the capacity to undertake commitment-bearing acts and to take responsibility for one’s words. The ACE framework generalizes this point. A work may display a stable “voice,” recurring evaluative preferences, or a recognizable perspective without thereby being the answerable expression of an author. To count as such, it must make available a standpoint attributable to an agent who can avow, revise, defend, or be challenged for it, and for which others may demand reasons or object to distortion and misattribution.

One might object that sufficiently sophisticated AI systems could do more than merely simulate a voice: they might genuinely manifest their own preferences or perspectives. Suppose, for example, that a system developed sui generis aesthetic preferences and expressed them consistently across its outputs. That possibility should not be excluded a priori. But even if such a system could be said to display expression in some meaningful sense, a further question would remain: whether that expression rises to the level required for authorship. Not every product of a minded being counts as authored expression. Accidental, automatic, insufficiently guided, or merely private outputs may issue from agents with beliefs and desires without thereby becoming authored works. What matters for authorship is not only that a work displays a recognizable voice, evaluative style, or perspective, but that this perspective be attributable to the putative author as a publicly attributable, commitment-bearing standpoint, one that can ground attribution, integrity, ownership, and the other normative incidents of authorship. Until that stronger condition is met, the presence of expression alone is not yet enough for authorship-grade expression.

Another objection might be that refusing to recognize authorship to AI systems reflects an unjustified “human exceptionalism” (Abbott, 2025: 665, see also Blaszczyk, 2025; Sarkar, 2023), a species-based bias that arbitrarily withholds a status to which AI systems are, functionally, entitled. Because several doctrines and court cases (see Sect.  2.1 and footnote 4) frame eligibility in explicitly human terms, exclusion can appear to rest on species membership rather than on any principled capacity of the agent.^15^ The point of translating the human-authorship rule into explicit capacity predicates in the ACE cluster is precisely to show that the relevant work is not done by biological membership as such. What copyright law implicitly tracks is a thicker agential profile: intentional agency, guided control, and relational expression. If current AI systems fail, they fail not because they are nonhuman, but because they have not been shown to satisfy that conjunction.

One virtue of the ACE framework is that it leaves the future open: if a nonhuman entity, including an AI system, were attributable with stable intentions of its own, exercised reasons-responsive guidance over a work’s formation, and occupied a public standpoint from which that work could count as its answerable expression, nothing in the framework would preclude recognizing it as an author. The present claim is narrower: current generative AI systems have not yet been shown to satisfy the thicker, responsibility-bearing conjunction of intentional agency, guided control, and relational expression that copyright authorship assumes.

The practical significance of this thicker account of authorship is that current legal disputes are already structured by it, even when courts and administrative bodies do not articulate it in these terms. In *Thaler v. Perlmutter*, no entity meets the intentional agency, control, and relational expression conditions for authoring *A Recent Entrance to Paradise*: there is therefore no author and the work is ineligible. In *Naruto v. Slater*, the macaque did not set out to take a photograph or control the process, triggering the shutter was accidental, and therefore the image falls under public domain. In mixed works such as *Zarya of the Dawn* (see footnote 17), the text and layout satisfy capacity predicates of authorship, while the raw Midjourney images do not, due to a lack of control from the user. Protection can therefore be granted only to the human contributions, with appropriate disclaimers, as current U.S. practice allows. These results apply, irrespective of substituting the human-authorship requirement with the ACE cluster. In this sense, current law does not simply require that the author is human. It presupposes a thicker, capacity-laden concept of authorship, of which the human-authorship rule is only a doctrinal proxy.

The next question, therefore, is how the law should allocate rights where AI assists, mediates, or displaces human creative activity, and what should happen when no candidate for authorship remains. The following section thus turns from conceptual reconstruction to legal design.

## Legal Design in AI Cases: Three Options

I turn now to considering three legal design options for allocating legal authorship in AI-related cases and how they fare in respect to the ACE cluster, copyright’s functions, and its doctrinal constraints: (1) recognizing AI as a legal author, (2) treating human users as authors of AI-assisted works when their contribution meets the ACE cluster threshold, and (3) accepting that some outputs are authorless and fall by default into the public domain.

### AI as Legal Author

The clearest and most prominent expression of this position is Thaler’s attempt to register *A Recent Entrance to Paradise* with the Creativity Machine listed as its author, a view also defended by legal scholars such as Abbott (2025) and Oleksy (2023). Assessed against the ACE framework as a reconstruction of the responsibility-bearing conception of authorship implicit in copyright law, this option is difficult to sustain on the present record. Recognizing AI as author would therefore require either showing that current systems satisfy the thicker, responsibility-bearing capacities that copyright authorship presupposes, or revising the conception of authorship built into copyright doctrine.

On the second axis–doctrinal consistency–recognizing AI systems as authors would be in conflict with most existing copyright statuses (as detailed in Sect.  2.1). In the U.S., it would conflict with the human-authorship requirement affirmed by the Copyright Office and by the courts. Similarly, in the EU, it would be in tension with the standard that requires the author’s “own intellectual creation.” Commonwealth provisions often invoked in AI debates assign authorship of computer-generated works to “the person by whom the arrangements necessary for the creation of the work are undertaken,” thereby preserving the human-author baseline, a baseline now explicitly endorsed in Italian copyright law, too. Under these circumstances, to treat AI as an author would require profound legislative change or reinterpretation. It would also raise significant administrability problems: even if AI were made author-eligible, the law would still need criteria for identifying which system is the author and for attributing outputs to that system across shifting versions, deployments, and human-AI production chains.

On the third axis–functional alignment–assigning copyright to AI systems would not directly promote copyright’s aims of rewarding labor and incentivizing creation for the public good. AI does not respond to incentives (Mazzi, 2024; *Thaler v. Perlmutter*).^16^ Any rights vested in them would be exercised, in practice, by the human or corporate entities that own or operate them. There is no reason to expect that attributing authorial status to AI would better motivate human innovation than attributing it to human designers or users would (Mammen et al., 2024). Moreover, assigning authorship to AI would lead to a risk of over-enclosure. The volume of content that can be generated by AI is potentially astronomical (Mazzi, 2024). If each output were protected by copyright, the public domain could be jammed with opaque rights, driving up transaction costs, stultifying creative practices (Mammen et al., 2024), and channeling control over cultural resources to a handful of technology firms (Craig & Kerr, 2019).

A related alternative would be to vest rights, by default, in the lawful owner of the model. In principle, that could be created as a legislative ownership-allocation rule, and Bridy (2012) is right that work-for-hire provides the closest existing template for such a move. But it is not available under current copyright law, because work-for-hire reallocates ownership only once there is a copyrightable work of authorship in the first place, it does not manufacture authorship where none exists (Thaler v. Perlmutter C.1; Ajani, 2022; Nawar, 2024, see also footnote 15). More fundamentally, ownership of a productive system is not the same as authorship of its outputs. Defaulting rights to model owners would therefore sever copyright from the responsibility-bearing contribution that authorship is meant to track, while raising the same over-enclosure and concentration concerns and doing little to advance copyright’s incentive rationale.

### Human Users as Authors of AI-Assisted Works

A more moderate proposal is to treat human users as authors of works generated with AI assistance, provided that they exercise sufficiently substantial control over the process of creation.

On the first axis, this option fits in principle the ACE cluster of authorship requirements. Someone who uses a system as a tool by supplying prompts, iterating on outputs, curating, selecting and editing them, can in principle satisfy intentional agency, control, and relational expression. Analogies from pre-AI practice are helpful here: photographers exercise authorship through staging, selection, and timing, even though the camera determines the exact distribution of light (*Burrow-Giles*, 111 U.S.; Caldwell, 2023), while collage and appropriation artists incorporate found or pre-existing materials and their authorship lies in how they select, frame, and recombine them intentionally and exercising creative control (Craig & Kerr, 2019; Irmak, 2024; Mazzi, 2024; Nawar, 2024).

The analogy is not perfect: AI systems are unlike cameras, as the control that the user can have on them through prompting or parameter selection is unlike the, arguably more direct, control that a photographer has on the camera settings. At the human–machine interface, the control that is available to human users is partial, and often insufficient to satisfy the threshold required for authorship. As Nawar argues, those capacities often do not include reliable parsing of elementary syntactic and compositional constraints so that “the AI’s output [is] insufficiently sensitive to the user’s input” (Nawar, 2024: 356). In such cases, the user lacks the control needed to make their reasons track into the work’s selection and arrangement of elements. Additionally, the process is not traceable to a specific agent’s capacity to do otherwise at the level that matters for authorship. It exhibits, in Santoni de Sio and Mecacci’s (2021) terms, a failure of tracking and tracing, a species of “responsibility gap,” transposed to authorship (Nawar, 2024).

These observations should not be mistaken for a denial that humans can exercise control through AI. They can, and often do, especially when systems are used as faithful instruments in realizing a pre-existing creative plan. The challenge is to determine when control exercised by the human user is enough to warrant for authorship. The ACE cluster can help to identify a principled threshold: authorship should be recognized when, and because, an agent’s intention, control, and expressive standpoint make an intelligible difference to the work as an *F*-work. That is a higher bar than one-click, uncurated generation, but lower than exhaustive control of every detail in the process. I acknowledge that the ACE cluster does not by itself resolve how to measure this threshold with precision. Operationalizing it will require further work to translate the principle into reliable, contestable decisions. Evidentiary practices would need to stabilize around what counts as reliable indicators of guided control and relational expression, for example records of prompting, parameter-setting, editing histories, and workflow documentation, and how such evidence is to be evaluated across different media and tools. In addition, AI systems and platforms change rapidly, which makes it harder to specify stable *ex ante* rules for disclosure. These constraints suggest that implementing this operationalization would require institutional guidance and compliance infrastructures in addition to doctrinal articulation.

The operationalization of the relevant threshold of control is a challenge that is currently being addressed by copyright doctrine. The U.S. Copyright Office’s March 2023 Copyright Registration Guidance makes explicit that “If a work’s traditional elements of authorship were produced by a machine, the work lacks human authorship and the Office will not register it.” (Copyright Registration Guidance, 88 Fed. Reg. at 16192) By contrast, human contributions are protectable where there is meaningful, intentional, creative control over expression. The Office’s inquiry is case-by-case and medium-specific: it asks how the tool operates, how it was used, and whether the human actually formed the expressive elements. Applicants must disclose any AI-generated content and identify the human authorship they claim and do not name the AI as author. Failure to disclose can lead to cancellation or a court disregarding the registration.^17^

On the axis of functional fit, attributing authorship to users has clear virtues. It directs incentives and rewards to human agents whose creative labor is involved (Mazzi, 2024). It preserves the connection between authorship and responsibility: users who claim authorship can be held to account for plagiarism, defamation, or harmful uses (Oleksy, 2024). The principal risk is over-extension: if thresholds are set too low, users may obtain monopolies over works in which their responsibility-bearing contribution is negligible, enclosing material that is largely the product of training-data corpora and generic prompts.

### Authorless AI Output

A third option is to accept that, where no contribution meets the ACE cluster of requirements, outputs have no author and the appropriate default is to treat such material as unprotected and part of the public domain.

Irmak’s discussion of “artifacts without authors” shows that there can be artefacts that fall under functional or structural kinds without any agent’s intentionality and responsibility underwriting their existence (Irmak, 2024). For example, in cases where an agent issues a generic prompt to a generative AI system and then selects one output for use, they may plausibly claim authorship of the selected item if they assume responsibility for its identity as a member of a kind. The unused items are “artifacts without authors,” they fall under artifact kinds (e.g., computer program, digital image) but there is no one who exercised the required control over the properties that make them members of those kinds (Irmak, 2024: 9–10).

On the doctrinal axis, the option fits existing U.S. practice: the Copyright Office has refused to register works “produced by a machine or mere mechanical process that operates randomly or automatically without any creative input or intervention from a human author.” (USCO 2021, § 313.2) In *Thaler v. Perlmutter*, the D.C. Circuit affirmed this stance by holding that a purely AI-generated image with no human authorship is ineligible and thereby falls under the public domain.

Ginsburg & Budiardjo’s (2019) central point about “authorless” outputs is that some machine-enabled works simply lack an author under copyright’s basic conception-plus-execution test. They claim this is particularly evident with partially generative systems: sometimes the user’s role is too thin, and the designer’s role is too upstream, so authorship doesn’t attach to either. The better default is to treat such outputs as public-domain material, unless and until there is strong evidence that a tailored, non-authorship regime, e.g., a sui generis right, is needed to supply incentives.

Functionally, leaving some AI-generated material in the public domain has clear advantages. It avoids conferring unmerited rights on users whose contributions are marginal and facilitates remixing and transformative uses that can feed back into further creations. The primary concern is incentives: would such a regime undercut investment in AI systems or AI-assisted creation? Here we should distinguish upstream (developers) and downstream (users) actors (Ginsburg & Budiardjo, 2019). Developers already enjoy protection for models via copyright of the source code, patents, trade secrets, and licensing agreements. Users are often motivated by non-copyright incentives: commission fees, reputational gains, or the possibility of using outputs as part of larger projects. There is little evidence that granting copyright to fully automated, un-curated outputs is necessary to incentivize either developers or users (Ginsburg & Budiardjo, 2019: 448). A practical difficulty is that “authorlessness” will often be contested at the margins. Implementing this option therefore depends on workable disclosure and proof practices capable of distinguishing genuinely authorless outputs from AI-assisted works in which a human contribution meets the relevant threshold.

## Conclusion

I have argued that copyright authorship is best understood as a responsibility-bearing status structured by intentional agency, guided control, and relational expression. The contribution of the paper is to make this underlying structure explicit, to show that the human-authorship rule is better read as a proxy for those capacities than as a simple species-based restriction, and to explain why recognizing current generative AI systems as authors sits uneasily with copyright’s foundations unless one is prepared to attribute to them the thicker responsibility-bearing capacities identified by ACE and copyright law. The paper also clarifies why some AI-assisted works may still qualify as human-authored, while some AI-generated outputs are better understood as giving rise to genuine authorship gaps.

Authorship is not only a legal category but also a public and moral one, closely associated with human creativity, expression, and accountability. A radical redefinition of authorship would likely require a broader shift in public attitudes. But not every reform requires a change of that magnitude. More modest measures, like clearer distinctions between AI-assisted and AI-generated works, stronger disclosure duties, or public-domain treatment for some outputs, could adapt copyright law to AI without abandoning the core features of our concept of authorship altogether. Operationalizing these measures will also require rule-of-law supports: workable evidentiary proxies for guided control, stable disclosure practices, and administrable standards that courts and registration authorities can apply consistently. At the same time, it is important to recognize how authorship is often distributed and mediated. The practical task I set for future work is to trace where ACE conditions are actually met in these networks and to make those contributions visible.

## Data Availability

Not applicable.

## Footnotes

1 I return in Sect.  3.1 to the conditions under which an agent counts as the author of a work that fits a specific sortal description.

2 Copyright law varies by jurisdiction. In this paper I focus primarily on U.S. copyright law, as it is most pertinent to current AI-related cases.

3 *Feist Publications v. Rural Telephone Service Co.* (1991) was a U.S. Supreme Court case about whether a phone book’s white pages were protected by copyright, with the Court holding that facts aren’t copyrightable and requiring at least a minimal degree of originality in selection or arrangement. Before *Feist*, many courts followed the “sweat of the brow” doctrine, effectively requiring no creativity.

4 The human-authorship requirement has been affirmed also in case law, most notably in *Urantia Foundation v. Maherra*, 114 F.3d 955, 958 (9th Cir. 1997); *Kelley v. Chicago Park Dist.*, 635 F.3d 290, 304 (7th Cir. 2011); *Naruto v. Slater*, 888 F.3d 418 (9th Cir. 2018).

5 Ireland, New Zealand, South Africa and India contain similar provisions (Abbott, 2025: 117–118).

6 Translation from the Italian by the author. In italics are the recent additions to the Article. Original text: “Sono protette ai sensi di questa legge le opere dell’ingegno *umano* di carattere creativo che appartengono alla letteratura, alla musica, alle arti figurative, all’architettura, al teatro ed alla cinematografia, qualunque ne sia il modo o la forma di espressione, *anche laddove create con l’ausilio di strumenti di intelligenza artificiale*,* purche’ costituenti risultato del lavoro intellettuale dell’autore*.”

7 Although there have been calls for copyright law to embrace a stronger vision of copyright holders’ responsibilities (Sun, 2013).

8 Link to the exhibition: https://www.nationalgalleries.org/exhibition/andy-goldsworthy-fifty-years.

9 For Elisabeth Anscombe what marks intentional action is that the question “Why?” (in the sense of asking for the agent’s reason) applies and can be answered by the agent with reason-giving answers (Anscombe, 1963: 9–12).

10 I used here an example from the arts, a field where the notion of authorship might differ from both legal and philosophical notions (Ginsburg & Budiardjo, 2019: 365). For example, many artistically central practices receive little or no copyright protection because they do not meet requirements such as fixation: live performance, improvised music, and ephemeral street art can be paradigm cases of artistic authorship, yet they do not fit neatly into the category of work of authorship “fixed in a tangible medium of expression.” (17 U.S. Code § 102(a), a doctrinal requirement for copyright protection.

11 In the case of “simpler” tools, like brush, cameras, or lights, this thin epistemic requirement of copyright is benign. In AI-mediated creation, however, the opacity and partial autonomy of systems can have repercussions on other conditions associated with authorship as a responsibility-bearing status. For example, inadvertent plagiarism and style appropriation may occur without the user’s knowledge, complicating duties to avoid plagiarism and respect others’ moral rights, and the labor of those who created the material that the training set consists of may be masked and uncredited, intensifying “many hands” issues and disputes over credit. For discussions on the “many hands” problem, see: Mühlhoff, 2025; Slota et al., 2023; Thompson, 1980, 2017; van de Poel et al., 2015.

12 This is one reason why AI raises somewhat different problems in patent law. Courts have accordingly held that AI systems cannot be listed as inventors under current U.S. patent law (*Thaler v. Vidal*, 43 F.4th 1207, 1210 (Fed. Cir. 2022), and recent scholarship on AI and patent doctrine has emphasized the distinctive role of conception and disclosure in inventorship (Abbott, 2016; Gibson, 2024; Mammen et al., 2024).

13 For further discussions on responsibility gaps, see: Danaher, 2022; Goetze, 2022; Gunkel, 2020; Königs, 2022; Hellström, 2013; Nyholm, 2018; Schur et al., 2025; Swaminathan & Danks, 2024.

14 AI-mediated creation in particular involves complex distributions of authorship and responsibility: users who exercise varying degrees of control, developers whose design choices shape the capabilities of the system, but also the often invisible or uncredited labor of crowd workers who label data, moderators who clean training sets, and the countless creators whose works are scraped to train models without acknowledgement (Shoemaker, 2024).

15 It is worth noting that the work-for-hire doctrine already recognizes “authors” that are not natural persons: the employer or commissioning entity is deemed the author in these cases. This doctrine has been indicated by some scholars as a possibly viable route to treat copyright and ownership attribution in cases involving AI (Bridy, 2012; Ginsburg & Budiardjo, 2019).

16 At least not to the incentives that copyright law refers to. Reward models that are employed in reinforcement learning would not be relevant here.

17 An example is the *Zarya of the Dawn* case, where the Office recognized human authorship in the comic’s text and the arrangement of images and words, but not in the raw Midjourney-generated images (Copyright Office, *Re: Zarya of the Dawn (Registration #VAu001480196)* (Feb. 21, 2023), https://perma.cc/AD86-WGPM).
